# Pointing Error Correction for a Moving-Platform Electro-Optical Telescope Using an Optimized Parameter Model

**DOI:** 10.3390/s23084121

**Published:** 2023-04-20

**Authors:** Yan He, Yahui Zhang, Xiaohua Feng, Shuwei Deng, Zhiwen Wang

**Affiliations:** 1 Institute of Optics and Electronics, Chinese Academy of Sciences, Chengdu 610209, China; heyan213@mails.ucas.ac.cn (Y.H.);; 2 School of Electronics, Electrical and Communication Engineering, University of Chinese Academy of Sciences, Beijing 100049, China; 3 University of Chinese Academy of Sciences, Beijing 100049, China; 4 Key Laboratory of Optical Engineering, Chinese Academy of Sciences, Chengdu 610209, China; 5 Chinese People’s Liberation Army Unit 95975, Lanzhou 732750, China

**Keywords:** moving-platform electro-optical telescope, pointing error, error correction, optimized parameter model

## Abstract

This paper proposes a new optimized parameter model that enhances the pointing accuracy of moving-platform electro-optical telescopes (MPEOTs). The study begins by comprehensively analyzing the error sources, including the telescope and the platform navigation system. Next, a linear pointing correction model is established based on the target positioning process. To eliminate multicollinearity, stepwise regression is applied to obtain the optimized parameter model. The experimental results show that the MPEOT corrected by this model outperforms the mount model, with pointing errors of less than 50 arcsec for approximately 23 h. In the three tests conducted, the modified azimuth error(s) (RMS) were 14.07″, 12.71″, and 28.93″, and the elevation error(s) (RMS) were 12.94″, 12.73″, and 28.30″, respectively.

## 1. Introduction

With the rapid advancement of photoelectric technology, electro-optical telescopes are increasingly being integrated into vehicles, ships, aircraft, and spacecraft for diverse missions, such as optical communication, aerial photography, astronomical observation, and antenna stabilization [1,2,3,4]. Control systems of moving-platform electro-optical telescopes (MPEOTs) require high-performance pointing directions to achieve accurate target acquisition, tracking, and positioning [5]. Once given the position of a target, the control system performs a series of coordinate transformations, which computes the angles necessary to point the telescope at the object. However, the actual line-of-sight (LOS) of MPEOT deviates inevitably from its desired pointing in practice [2], namely a pointing error, which impairs the efficiency of the target acquisition and tracking as well as the imaging quality. Therefore, to improve the MPEOT’s overall performance, error modeling and pointing correction techniques should be investigated thoroughly.

Errors affecting pointing arise during the manufacture, assembly, installation, and operation of the telescope, including machining errors in components, geometrical variations induced by misalignment and improper installation, inaccuracy of encoders, servo errors, environmental errors, etc. [6]. In general, errors from a particular source have two components: a deterministic part and a random part [7]. The paper contributes to understanding the calibration of the repeatable systematic errors, while the remaining nonlinear errors are not covered in this work.

Pointing error correction is generally classified into two types: hardware calibration and software correction [8]. The former acquires the angular deviation and coordinate transformation matrix between the components using external devices, such as calibration methods with the use of theodolites [9,10] and other measuring equipment [11], so as to be compensated in the control system. However, the resources needed are significant and not necessarily effective. Based on error analysis and modeling, the latter estimates the systematic errors and deducts them from the current pointing value in real time, then it updates position commands that control the telescope axes to compensate for pointing errors. In contrast to hardware adjustment, software rectification significantly increases pointing accuracy while reducing the development and cost; it is the research focus of this article.

Considerable progress has been made in the field of error modeling by directly considering parameters that are highly related to performance. Tan et al. investigated the relationship between pointing error and temperature distribution for inter-satellite communication [12]. In some optical communication systems, pointing errors are avoided or reduced by focusing on system-wide performance metrics, such as the received signal power and Strehl ratio [13]. Lopez-Leyva et al. emulated the effects of pointing error angles on the signal-to-noise ratio to optimize the pointing process [14]. For ground-based telescopes, the pointing error model can be expressed traditionally as a nonlinear function of azimuth and elevation [15], which can be further divided into two categories. One approach relies solely on numerical analysis and adopts an empirical model to explain the observed differences. A single function can be used to fit the error–correction surface across the entire sky or the sky can be broken down into regions and different functions used for each, such as the spherical harmonics function model and generalized extended approximation model [16,17]. Analogously, Zhu [18] and Xu [19] proposed the use of the backpropagation (BP) neural network model and radial basis function neural network model, respectively, tested and applied in the mobile laser ranging station. Although this strategy is simple to apply, the stability of the model is poor due to excessive parameters and the high correlation between them. Another approach involves studying the physical relationships underlying pointing errors and developing a model about them, including the basic parameter model, mount model, and physical model with empirical terms [20,21,22]. Many telescopes calibrated by this method point to the arcsec level of precision [23,24,25]. Such models typically have fewer terms and correspond to physical errors within the telescope. Additionally, this practice is more likely to provide reliable outcomes when used to extrapolate outside of the scope of the sky that the model was initially fit [7].

However, the technique above-used for ground-based telescopes is not fully functional for MPEOT due to platform vibration and environmental disturbance. Currently, the most effective way to obtain pointing error models is by observing a group of stars in the sky region to acquire the pointing error data of the telescope at each position, including selecting stars, building mathematical models, and solving parameters [15,16,17,18,19,20,21,22,23,24,25,26,27]. The model only validly works for a while. As time passes, the physical properties and environment of the telescope change, requiring a new model to be built by re-observing the stars. It is worth noting that MPEOTs require long exposures in target observations, during which residuals revised by the above models tend to fluctuate widely. This phenomenon indicates that the model parameters are time-limited, and a single correction cannot maintain the high accuracy of the MPEOT pointing over time. As a countermeasure, this paper proposes a new optimized parameter model based on the physical kinematic model and the correlation characteristics of the parameters, which can significantly mitigate the pointing error during a longer working period.

The remaining part of the essay proceeds as follows. Section 2 reinterprets some operating procedures and correction guidelines. Section 3 summarizes the error sources and extracts a linear pointing correction model directly from the target localization equation. Section 4 describes the multicollinearity problem between parameters, using stepwise regression to optimize the linear model. To verify the validity of the optimized model, several experiments are conducted in Section 5. The relevant discussion and conclusions on the experimental results are presented in Section 6 and Section 7, respectively.

## 2. Operation Principle

### 2.1. EOT Structure

**Figure 1:**
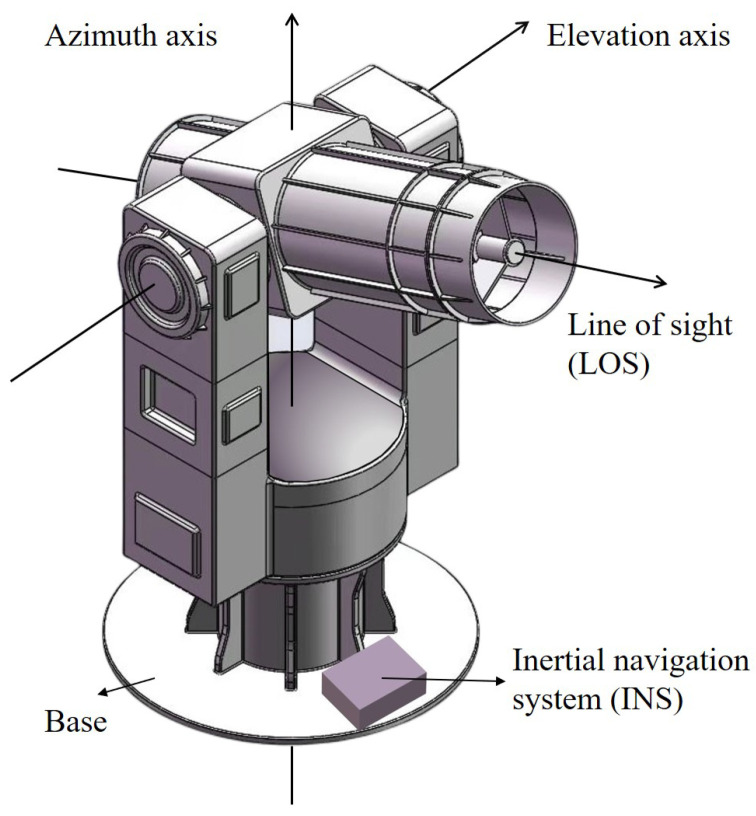
Gimbal arrangement of the electro-optical telescope.

The components of an electro-optical telescope (EOT) can be typically divided into functional subsystems that contribute to understanding the errors that affect pointing [26,27]. The electro-mechanical gimbal, consisting of a base, bearings, motors, actuators, etc., provides physical support and allows the motion of the axes. This paper concentrates on the alt-azimuth telescope [28], whose gimbal arrangement is depicted in Figure 1, including the azimuth axis, elevation axis, and line of sight. The servo control system generates and coordinates the motion, while the optical system collects and transmits information about the targets.

### 2.2. Correction Principle

**Figure 2:**
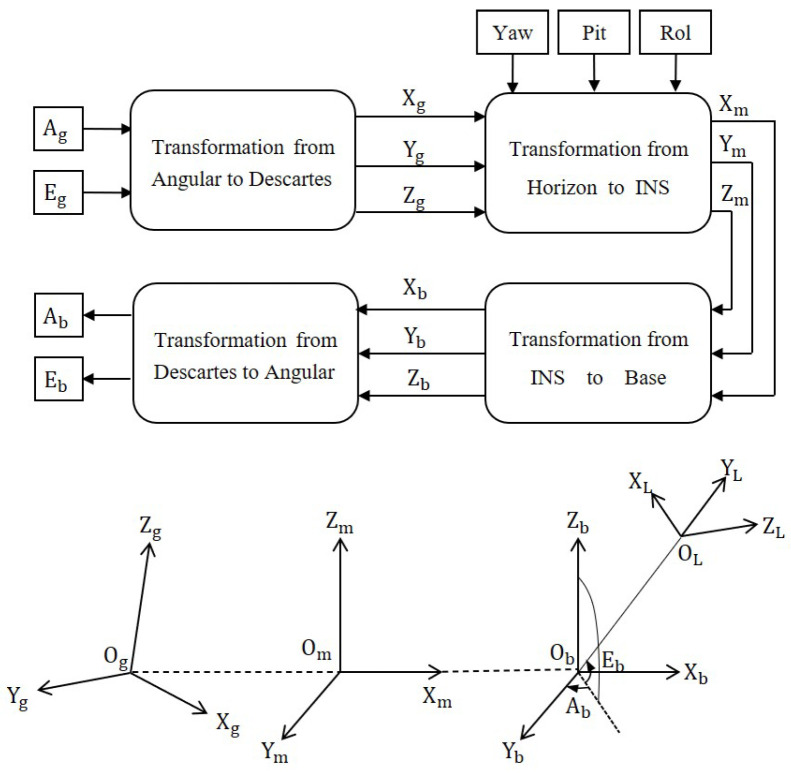
Target pointing process of EOT.

In Figure 2, the procedure for calculating the desired pointing in the MPEOT system is displayed. For further elaboration, several left-hand coordinate frames are defined first, which are the geographic coordinates *g*, moving-platform coordinate *m*, telescope base coordinate *b*, and target coordinate *L*. Based on the theoretical pointing angle $\left(A_{g},E_{g}\right)$ of the target in the geographic coordinate, the control system can then calculate the expected direction $\left(A_{b},E_{b}\right)$. Therefore, the target positioning equation of EOT can be formulated as

(1) $$G=R_{m}^{g}R_{b}^{m}=B$$

where $G={\left[X_{g},Y_{g},Z_{g}\right]}^{T},B={\left[X_{b},Y_{b},Z_{b}\right]}^{T},R$ is the rotation transformation matrix between the coordinate frames. In an ideal condition, the azimuth axis, the elevation axis, and the line of sight are three perpendicular axes in zero-elevation positions [28]. In this case, the pointing model of the telescope can be defined as

(2) $$B=R_{L}^{b}L=Rot\left(Z,A_{b}\right)Rot\left(X,E_{b}\right)L$$

Assuming that $L={\left[0,1,0\right]}^{T}$ is the unit pointing direction referenced to the target coordinate, the pointing angle of the telescope can be determined from Equation (2) and expressed in the base coordinate as

(3) $$\left\{\begin{array}{c} A_{b}=\arctan X_{b}/Y_{b}\\ E_{b}=\arcsin Z_{b} \end{array}\right.$$

**Figure 3:**
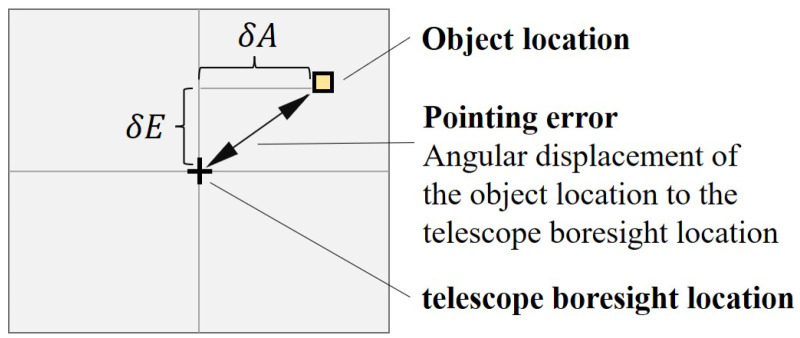
Definition of pointing error.

The pointing error is defined as the angular displacement with which the pointing control system positions the actual object relative to its telescope boresight location after acquiring a suitable target for observation [29]. As illustrated in Figure 3, the angular pointing errors of the azimuth and elevation axes of the telescope can be denoted as δA and δE [27], respectively. They are given by

(4) $$\left\{\begin{array}{c} \delta A=\hat{A}-A_{b}=f\left(\cdot \right)\\ \delta E=\hat{E}-E_{b}=g\left(\cdot \right) \end{array}\right.$$

where $\hat{A}$ and $\hat{E}$ are the actual azimuth and elevation angles in the base coordinate while $A_{b}$ and $E_{b}$ are the theoretical pointing angles. f(·) and g(·) represent error models for azimuth and elevation angles, respectively. In particular, both δA and δE contain the error mean and standard deviation, hence the pointing error is normally characterized using the root mean square (RMS) [30].

Thus far, the correction of EOT pointing errors entails the following steps. (1) Developing a relatively accurate and complete pointing error correction model using error source analysis. (2) Given a series of reference stars with known coordinates, the pointing errors can be calculated from the above equations by the measured values of EOT. (3) Estimating the parameters in Formula (4) and optimizing the correction model in reverse using the results. (4) Predicting and correcting the pointing error of the telescope using the solved model.

## 3. Error Analysis and Pointing Model

### 3.1. Sources of Pointing Error

**Figure 4:**
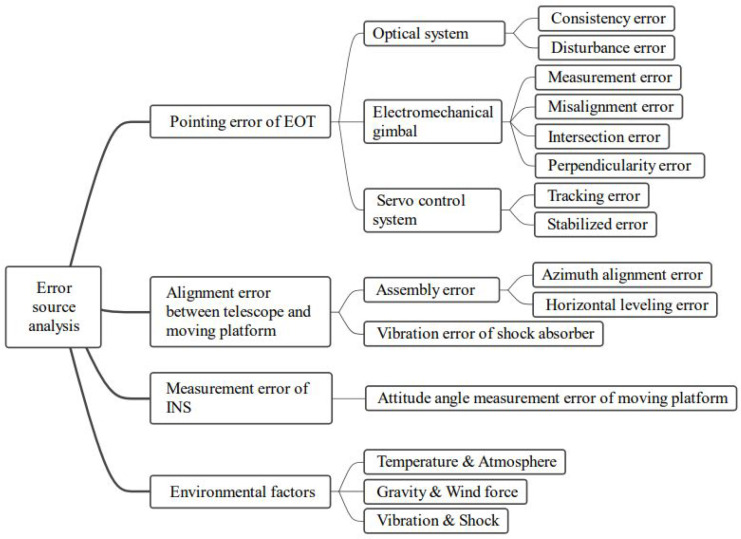
Error source analysis.

According to Figure 4, the pointing error sources of MPEOT can be categorized into four main groups [31]. Firstly, equipment-related pointing errors include (1) errors of the optical system, mainly caused by disturbance and inconsistency between the boresight of various optical components, (2) errors of electro-mechanical gimbals, which refer to geometric errors between axes, such as perpendicularity error, intersection error, misalignment error, and readout transducer error, and (3) errors of the servo control system, specifically the tracking error and stability error. Secondly, misalignment between the telescope base and the platform navigation system can be classified into two types: (1) orientation alignment error and horizontal leveling error during initial setup, and (2) angular vibration error during the operation. Next, the inaccuracy of the platform navigation system, notably the attitude measurement error of the inertial navigation system (INS), should be considered. Meanwhile, factors such as temperature, gravity, wind, air pressure, vibration, and shock can also have different effects on the above errors. To unify the coupling relationship between the static and dynamic errors of the system, this dissertation delineates several error coordinate frames in detail, derives their coordinate transformation matrices, and then establishes a telescope-pointing model.

### 3.2. Coordinates Definition and Transformation

As shown in Figure 2, the error conversion matrix from the motion platform navigation system coordinate *m* to the geographic coordinate *g* is

(5) $${\hat{R}}_{g}^{m}=Rot\left(Y,Rol+\Delta r\right)Rot\left(X,Pit+\Delta p\right)Rot\left(Z,Yaw+\Delta y\right)$$

where Rol,Pit, and Yaw are the roll, pitch, and azimuth angles respectively, serving as the real attitudes of the platform, and Δr,Δp,Δy are the corresponding measurement errors. The orientation-sensitive axis of the INS and the zero position of the base can be normally aligned by a right-angle prism mounted on the device, and the pitch and roll can be obtained from a leveling instrument, so it is reasonable to assume that the initial installation error matrix is known, here set to $R_{b1}$. Moreover, supposing that the transformation matrix between the base coordinate and the platform coordinate is $R_{b2}$ when the platform is in motion. Accordingly, the alignment error between *b* and *m* can be simply set as

(6) $${\hat{R}}_{b}^{m}=R_{b1}R_{b2}=R_{b1}Rot\left(Y,\Delta w\right)Rot\left(X,\Delta x\right)Rot\left(Z,\Delta z\right)$$

where Δw,Δx,Δz are angular vibration errors along each axis direction.

**Figure 5:**
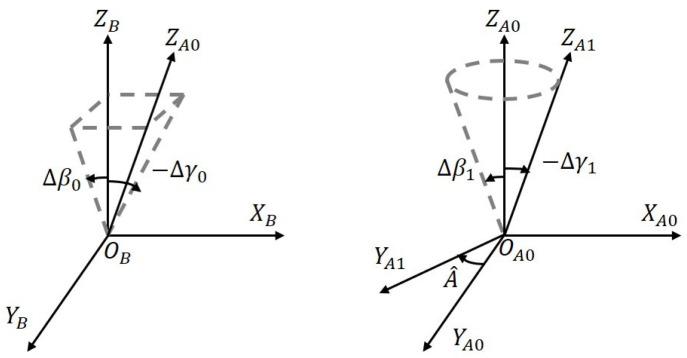
Shafting errors of the azimuth axis.

The shafting error of the azimuth axis relative to the base, demonstrated in Figure 5, mainly comprises the geometric frame error, servo control system error, and encoder error. Several coordinates are involved, defined as follows: (1) The base coordinate *b*, whose $X_{B}$ and $Y_{B}$ axes are in the mounting plane of the telescope and the moving platform, while the $Z_{B}$ axis is perpendicular to that plane. (2) The azimuth base coordinate A0, whose $Y_{A0}$ axis is parallel to the zero direction of the azimuth encoder and the $Z_{A0}$ axis is perpendicular to the outer flange plane of the azimuth frame. (3) The azimuth gimbal coordinate A1, which is attached to the azimuth frame and used to describe the azimuth axis movement. The transformation matrix of the azimuth axis can be expressed as

(7) $$\left\{\begin{array}{c} {\hat{R}}_{B}^{A0}=Rot\left(Y,-\Delta \gamma_{0}\right)Rot\left(X,\Delta \beta_{0}\right)\\ {\hat{R}}_{A0}^{A1}=Rot\left(Y,-\Delta \gamma_{1}\right)Rot\left(X,\Delta \beta_{1}\right)Rot\left(Z,\hat{A}\right) \end{array}\right.$$

$${\hat{R}}_{B}^{A0}=\left[\begin{array}{ccc} \cos(\Delta \gamma_{0}) & 0 & \sin(\Delta \gamma_{0})\\ 0 & 1 & 0\\ -\sin(\Delta \gamma_{0}) & 0 & \cos(\Delta \gamma_{0}) \end{array}\right]\left[\begin{array}{ccc} 1 & 0 & 0\\ 0 & \cos(\Delta \beta_{0}) & \sin(\beta_{0})\\ 0 & -\sin(\beta_{0}) & \cos(\beta_{0}) \end{array}\right]$$

$$Rot\left(Z,\hat{A}\right)=\left[\begin{array}{ccc} \cos(\hat{A}) & -\sin(\hat{A}) & 0\\ \sin(\hat{A}) & \cos(\hat{A}) & 0\\ 0 & 0 & 1 \end{array}\right]$$

where ($\Delta \gamma_{0},\beta_{0}$) denotes the perpendicularity error, $\Delta \gamma_{1},\Delta \beta_{1}$ denotes the rotation error, $\hat{A}=A_{b}+\Delta A=A_{b}+\Delta A_{s0}+\Delta A_{s1}+\Delta A_{s2}$, $A_{b}$ denotes the desired azimuth, $\Delta A_{s0}$ denotes the zero position error, $\Delta A_{s1}$ denotes the tracking error, and $\Delta A_{s2}$ denotes the encoder measurement error.

**Figure 6:**
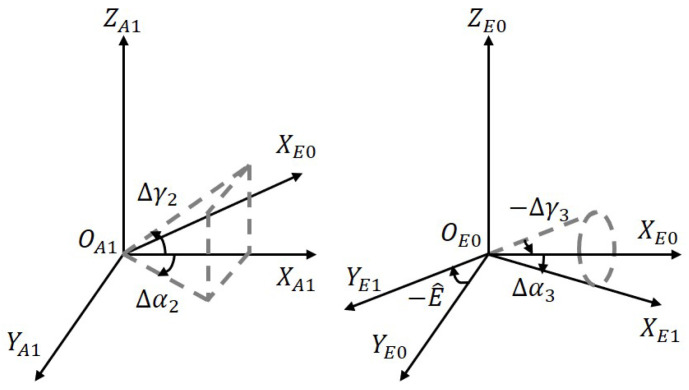
Shafting errors of the elevation axis.

Similar to the azimuth axis, Figure 6 illustrates the elevation axis shafting errors. The azimuth gimbal coordinate A1, elevation base coordinate E0, and elevation gimbal coordinate E1 are involved. The transformation matrix from the azimuth gimbal A1 to the elevation gimbal E1 can be expressed as

(8) $$\left\{\begin{array}{c} {\hat{R}}_{A1}^{E0}=Rot\left(Y,\Delta \gamma_{2}\right)Rot\left(Z,\Delta \alpha_{2}\right)\\ {\hat{R}}_{E0}^{E1}=Rot\left(Y,-\Delta \gamma_{3}\right)Rot\left(X,\hat{E}\right)Rot\left(Z,\Delta \alpha_{3}\right) \end{array}\right.$$

where $\hat{E}=E_{b}+\Delta E=E_{b}+\Delta E_{s0}+\Delta E_{s1}+\Delta E_{s2}$.

**Figure 7:**
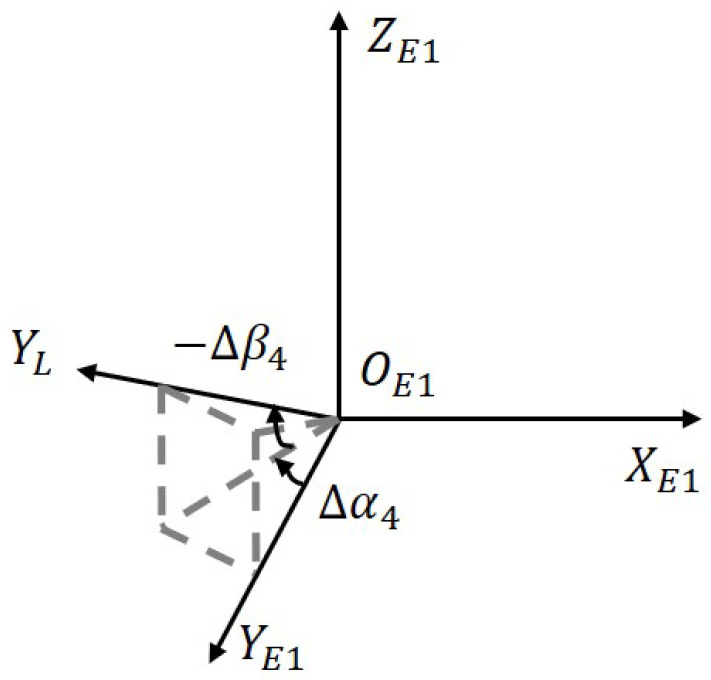
Perpendicularity error of optical axis.

Figure 7 portrays the optical axis perpendicularity error between the target coordinate *L* and the elevation coordinate *E*. Due to the two-axis frame of the telescope, there is no relative movement of the optical axis with respect to the elevation axis, implying no rotation error. Therefore, the error transformation matrix is expressed as

(9) $${\hat{R}}_{E1}^{L}=Rot\left(X,-\Delta \beta_{4}\right)Rot\left(Z,\Delta \alpha_{4}\right)$$

### 3.3. Linear Model Construction

In summary, the actual positioning equation of the observed target from the geographic coordinate *g* to the target coordinate *L* can be obtained based on Formulas (5)–(9).

(10) $$\hat{G}={\hat{R}}_{m}^{g}{\hat{R}}_{B}^{m}{\hat{R}}_{A0}^{B}{\hat{R}}_{A1}^{A0}{\hat{R}}_{E0}^{A1}{\hat{R}}_{E1}^{E0}{\hat{R}}_{L}^{E1}\hat{L}$$

Accordingly, in practice, the pointing error model of the MPEOT is

(11) $$\left\{\begin{array}{c} B_{1}=\hat{G}{\hat{R}}_{m}^{g}{\hat{R}}_{b}^{m}\\ B_{2}={\hat{R}}_{A0}^{b}{\hat{R}}_{A1}^{A0}{\hat{R}}_{E0}^{A1}{\hat{R}}_{E1}^{E0}{\hat{R}}_{L}^{E1}\hat{L} \end{array}\right.$$

where $\hat{G}={\left[X_{g},Y_{g},Z_{g}\right]}^{T},\hat{L}={\left[0,1,0\right]}^{T},B_{1}={\left[X_{b1},Y_{b1},Z_{b1}\right]}^{T},B_{2}={\left[X_{b2},Y_{b2},Z_{b2}\right]}^{T}$. To sequentially expand Equation (11), using the following simplification principle, each error factor Δ is a small quantity, i.e., cosΔ=1,sinΔ=0 while ignoring the higher order error term.

(12) $$\left\{\begin{array}{c} X_{b1}=k_{x}+m_{y}\Delta y+m_{p}\Delta p+m_{r}\Delta r+m_{z}\Delta z+m_{w}\Delta w\\ Y_{b1}=k_{y}+n_{y}\Delta y+n_{p}\Delta p+n_{z}\Delta z+n_{x}\Delta x\\ Z_{b1}=k_{z}+h_{y}\Delta y+h_{p}\Delta p+h_{r}\Delta r+h_{x}\Delta x+h_{w}\Delta w \end{array}\right.$$

(13) $$\left\{\begin{array}{c} \begin{array}{cc} X_{b2} & =\Delta_{y0}\sin\left(E_{b}\right)-\left(\Delta \beta_{4}+\Delta E-\Delta \beta_{1}\right)\sin\left(A_{b}\right)\sin\left(E_{b}\right)+\Delta \alpha_{4}\cos\left(A_{b}\right)\\ & +\cos\left(E_{b}\right)\sin\left(A_{b}\right)+\left(\Delta A+\Delta \alpha_{2}+\Delta \alpha_{3}\right)\cos\left(A_{b}\right)\cos\left(E_{b}\right)\\ \; & +\left(\Delta \gamma_{1}-\Delta \gamma_{2}\right)\sin\left(A_{b}\right)\sin\left(E_{b}\right)\\ Y_{b2} & =\Delta \beta_{0}\sin\left(E_{b}\right)-\Delta \alpha_{4}\sin\left(A_{b}\right)-\left(\Delta \beta_{4}+\Delta E-\Delta \beta_{1}\right)\cos\left(A_{b}\right)\sin\left(E_{b}\right)\\ & +\cos\left(A_{b}\right)\cos\left(E_{b}\right)-\left(\Delta A+\Delta \alpha_{2}+\Delta \alpha_{3}\right)\cos\left(E_{b}\right)\sin\left(A_{b}\right)\\ & -\left(\Delta \gamma_{1}-\Delta \gamma_{2}\right)\sin\left(A_{b}\right)\sin\left(E_{b}\right)\\ Z_{b2} & =\left(\Delta \beta_{1}-\Delta E\right)\cos\left(E_{b}\right)-\sin\left(E_{b}\right)-\Delta \beta_{4}\cos\left(E_{b}\right)\\ \; & +\Delta \beta_{0}\cos\left(A_{b}\right)\cos\left(E_{b}\right)+\Delta \gamma_{0}\cos\left(E_{b}\right)\sin\left(A_{b}\right) \end{array} \end{array}\right.$$

In addition, the conversion of Cartesian coordinates into pointing angles is given by

(14) $$\left\{\begin{array}{c} \tan\left(A_{b}+\delta A_{1}\right)={\hat{X}}_{b1}/{\hat{Y}}_{b1}\\ \sin\left(E_{b}+\delta E_{1}\right)={\hat{Z}}_{b1} \end{array}\right.\left\{\begin{array}{c} \tan\left(A_{b}+\delta A_{2}\right)={\hat{X}}_{b2}/{\hat{Y}}_{b2}\\ \sin\left(E_{b}+\delta E_{2}\right)={\hat{Z}}_{b2} \end{array}\right.$$

Substituting Equations (12) and (13) into Equation (14) and expanding, a linear model of the pointing error, named the full parameter model (FPM), is obtained as

(15) $$\left[\begin{array}{c} \delta A\\ \delta E \end{array}\right]=\left[\begin{array}{c} \delta A_{1}+\delta A_{2}\\ \delta E_{1}+\delta E_{2} \end{array}\right]=HX+\left[\begin{array}{c} \varepsilon_{A}\\ \varepsilon_{E} \end{array}\right]$$

$$H^{T}=\left[\begin{array}{cc} \Delta \beta_{0} & \Delta \beta_{0}\\ \Delta \gamma_{0} & \Delta \gamma_{0}\\ \left(\Delta \gamma_{1}-\Delta \gamma_{2}\right) & \Delta \gamma \\ \Delta \alpha_{4} & \Delta p\\ \Delta \gamma & \Delta r\\ \Delta p & \Delta x\\ \Delta r & \Delta w\\ \Delta z & \Delta \beta_{1}-\Delta E-\Delta \beta_{4}-2\tan\left(E_{b}\right)+k_{E2}\\ \Delta x & 0\\ \Delta w & 0\\ \Delta A+\Delta \alpha_{2}+\Delta \alpha_{3}+k_{A2} & 0 \end{array}\right],X=\left[\begin{array}{cc} p_{1} & q_{1}\\ p_{2} & q_{2}\\ p_{3} & q_{3}\\ p_{4} & q_{4}\\ p_{5} & q_{5}\\ p_{6} & q_{6}\\ p_{7} & q_{7}\\ p_{8} & 1\\ p_{9} & 0\\ p_{10} & 0\\ 1 & 0 \end{array}\right].$$

$\begin{array}{c} p_{1}=-\sin\left(A_{b}\right)\tan\left(E_{b}\right),p_{2}=\cos\left(A_{b}\right)\tan\left(E_{b}\right),p_{3}=\tan\left(E_{b}\right),p_{4}=\sec\left(E_{b}\right),\\ p_{5}=\frac{m_{y}\cos\left(A_{b}\right)-n_{y}\sin\left(A_{b}\right)}{k_{fm}},p_{6}=\frac{m_{p}\cos\left(A_{b}\right)-n_{p}\sin\left(A_{b}\right)}{k_{fm}},p_{7}=\frac{m_{r}\cos\left(A_{b}\right)}{k_{fm}},p_{8}=\frac{m_{z}\cos\left(A_{b}\right)-n_{z}\sin\left(A_{b}\right)}{k_{fm}},\\ p_{9}=-\frac{n_{x}\sin\left(A_{b}\right)}{k_{fm}},p_{10}=\frac{m_{w}\cos\left(A_{b}\right)}{k_{fm}},k_{A2}=\frac{k_{x}\cos\left(A_{b}\right)-k_{y}\sin\left(A_{b}\right)}{k_{fm}},k_{fm}=k_{y}\cos\left(A_{b}\right)+k_{x}\sin\left(A_{b}\right),\\ q_{1}=\cos\left(A_{b}\right),q_{2}=\sin\left(A_{b}\right),q_{3}=h_{y}\sec\left(E_{b}\right),q_{4}=h_{p}\sec\left(E_{b}\right),q_{5}=h_{r}\sec\left(E_{b}\right),\\ q_{6}=h_{x}\sec\left(E_{b}\right),q_{7}=h_{w}\sec\left(E_{b}\right),k_{E2}=k_{z}\sec\left(E_{b}\right)-tan\left(E_{b}\right). \end{array}$

## 4. Model Optimization Based on Stepwise Regression

### 4.1. Multicollinearity

A multiple linear regression model $y=\beta_{0}+\beta_{1}x_{1}+\beta_{2}x_{2}+\ldots +\beta_{p}x_{p}$ has a basic assumption that is rank (*X*) =p+1, where *X* is known as the design matrix. If there exist *p* numbers, making the following equation true, multicollinearity exists between the independent variables ($x_{1},x_{2},\ldots ,x_{p}$) [32].

(16) $$c_{0}+c_{1}x_{i1}+c_{2}x_{i2}+\ldots +c_{p}x_{ip}\approx 0,i=1,2,\ldots ,n$$

Objectively speaking, when an event involves multiple factors, there is typically a certain degree of correlation between them. When a group of variables is strongly correlated with each other, it is considered to violate the basic assumption of the multiple linear regression model. In Equation (15), the terms of the full parameter model are trigonometric functions of the angular positions of the axes, which are interrelated with each other. Therefore, it is reasonable to infer that the explanatory variables of this model have significant collinearity.

If the regression model has multicollinearity, the parameter estimates via OLSE are particularly unstable, and the variance of the coefficients rises more rapidly as the multicollinearity obtains more. This effect can result in a situation where some independent variable regression coefficients fail significance tests when the regression equation is highly significant, and the structural relationships between variables become distorted. Although multicollinearity has no influence on data fitting, it is worth noting that if the correlation of independent variables cannot be guaranteed to remain static in the prediction period, multicollinearity will have a significant negative effect on the regression prediction and misrepresent the prediction results.

A vital technique for assessing the degree of multicollinearity and diagnosing is the variance inflation factor (VIF) methodology [33]. Let $X^{{*}^{\prime }}X^{*}=\left(r_{ij}\right)$ be the correlation matrix of the independent variables after central standardization, and then the diagonal elements of its inverse matrix are the variance inflation factors $VIF_{j}=c_{jj}$. The formula is as follows.

(17) $$C=\left(c_{ij}\right)={\left(X^{{*}^{\prime }}X^{*}\right)}^{-1}$$

It is proven that the $c_{jj}$ and the coefficient of multiple determination $R_{j}^{2}$ are related:

(18) $$c_{jj}=\frac{1}{1-R_{j}^{2}}$$

The stronger the linear correlation (measured by the $R_{j}^{2}$) of the independent variable $x_{j}$ with the others, the more severe multicollinearity is. It is empirically shown that there is serious multicollinearity between the independent variables when $VIF_{j}\geq 10$, which might greatly affect the least-squares estimates [32].

### 4.2. Stepwise Regression

**Figure 8:**
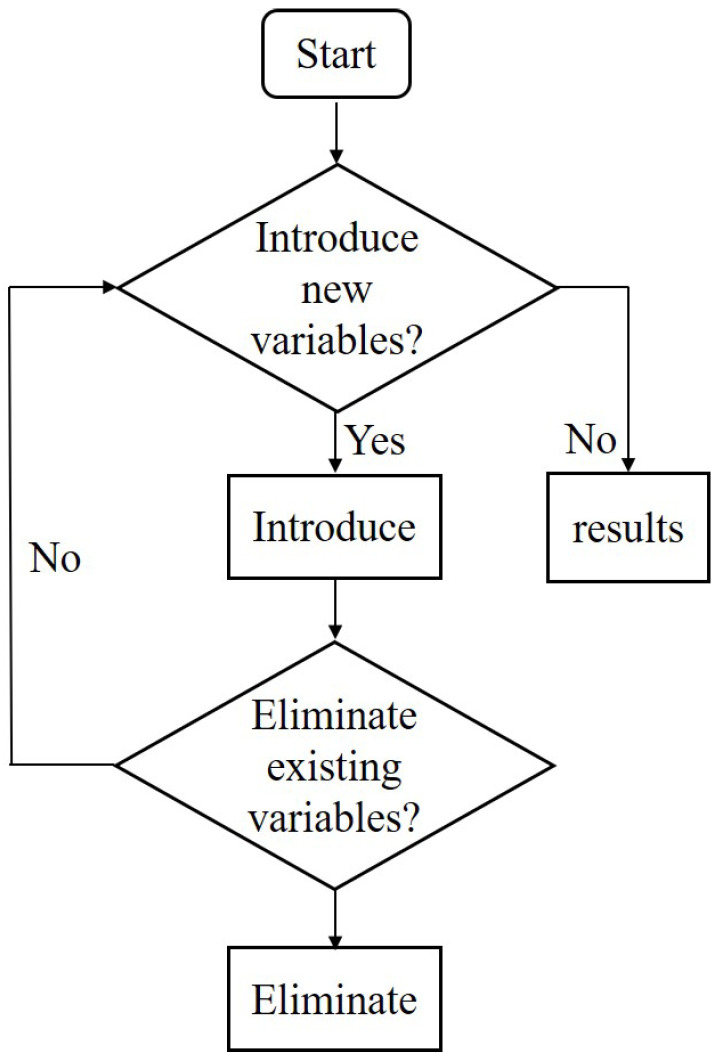
Schematic diagram of the stepwise regression process.

The most common approach to eliminate multicollinearity is to re-select the independent variables, including the forward method, the backward method, and the most commonly used stepwise regression [32]. As shown in Figure 8, variables are introduced sequentially and required to be removed when the original variables no longer become significant due to the later ones. Notably, only one variable can be introduced or excluded at a time. Furthermore, to guarantee that only significant variables are incorporated into the regression equation, an F-test is essential for each procedure. This operation continues until no more variables are imported or deleted. That is, the idea of stepwise regression is essentially in and out, making it possible to introduce the elements that strongly relate to the dependent variable first and then eliminate those with overlapping contributions. In short, the stepwise regression compensates for the defects of the forward and backward methods, making the final regression subset optimal.

### 4.3. Evaluation of Optimized Parameter Model

Properly dropping insignificant independent variables can considerably reduce or even eliminate the multicollinearity of the regression model. Moreover, the following common criteria can be used to measure the merit of the subset selection [32]. (1) Maximize the freedom-adjusted multiple determination coefficients $R_{a}^{2}$. This principle only pursues the goodness of fit of the data. Assuming that *n* and *p* are the numbers of samples and independent variables, respectively, with the following specific expression.

(19) $$R_{a}^{2}=1-\frac{n-1}{n-p-1}\left(1-R^{2}\right)$$

(2) Akaike information criterion (AIC). The model that matches this criterion provides the best explanation for the data but has the least free parameters. Given that the random error term of the regression model obeys a normal distribution, the likelihood function leads to the equation.

(20) AIC=nln(SSE)+2p

(3) Minimize statistics $C_{p}$. Mallows (1964) proposed a statistic that can be used for selecting independent variables in terms of prediction.

(21) $$C_{p}=\left(n-m-1\right)\frac{SSE_{p}}{SSE_{m}}-n+2p$$

In particular, *m* and *n* refer to the number of independent variables of the whole model and the selected model, respectively.

The priority of the criteria selection is different depending on the purpose of the research. If the regression equation is intended for forecasting, the $C_{p}$-statistic criterion is first considered, which minimizes the mean square error of the predicted values. In reality, the criteria should be considered comprehensively.

## 5. Experiment and Results

Having discussed how to construct the optimized parameter model (OPM) of the pointing error based on stepwise regression, this section conducts star-gazing experiments and evaluates the effectiveness of the model proposed in this paper compared to the full parameter model (FPM) and the traditional mount model (MM). An electro-optical telescope carried out 4 observations of stars randomly distributed throughout the sky within 2 days, in which researchers collected a total of 54 valid data sets. Moreover, during each observation, the telescope mounted on a sailing ship pointed intermittently to a star and made 10 samples per ‘pointing’.

### 5.1. Data Preparation

**Figure 9:**
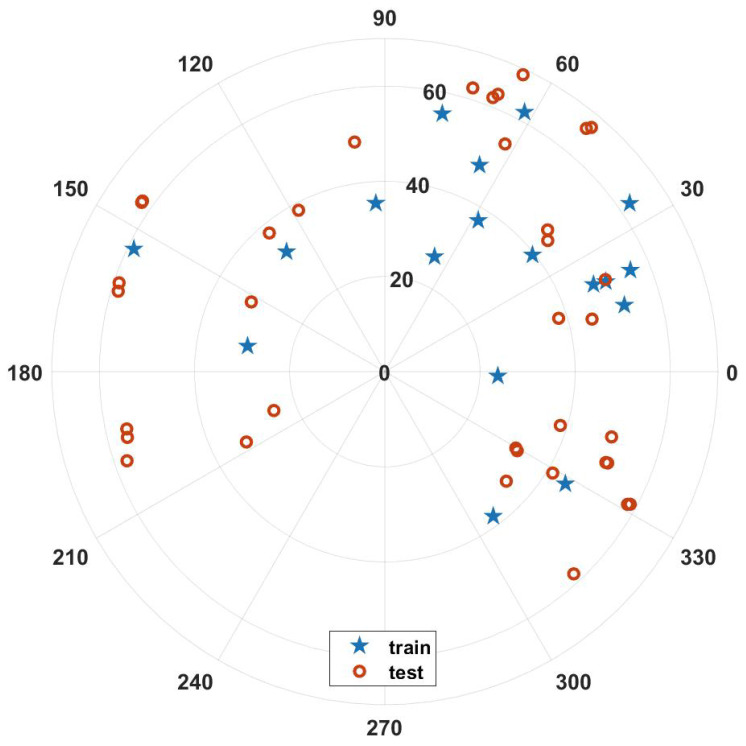
Star map distribution of the experiment.

In order to assess the fitting effect of the error model and the temporal validity of the parameters, the 18 sets of data collected from the first observation were used as training samples and the data gathered from the remaining 3 observations were used as test samples in this work. The distribution of the theoretical positions of the satellites in geographic coordinates during the observation period is shown in Figure 9, where the test samples cover an area partially beyond that of the training samples.

**Figure 10:**
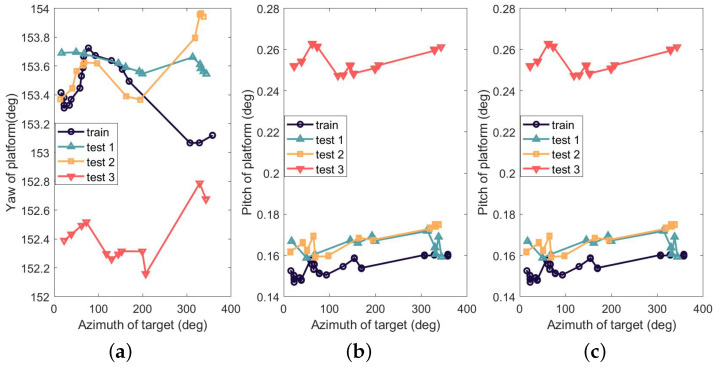
Attitude of moving-platform (**a**) in Yaw (**b**) in Pitch (**c**) in Roll.

Figure 10 depicts the attitude of the moving platform during the observations; aside from the first 18 pointing tests, the rest only contain 12 sets of data per test. It is obvious that the attitude of the platform in the last test is different from the previous ones. This difference is logical given that the first three observations begin at 20:00, 22:13, and 22:19 of the same day, while the last observation starts at 19:23 of the next day.

**Figure 11:**
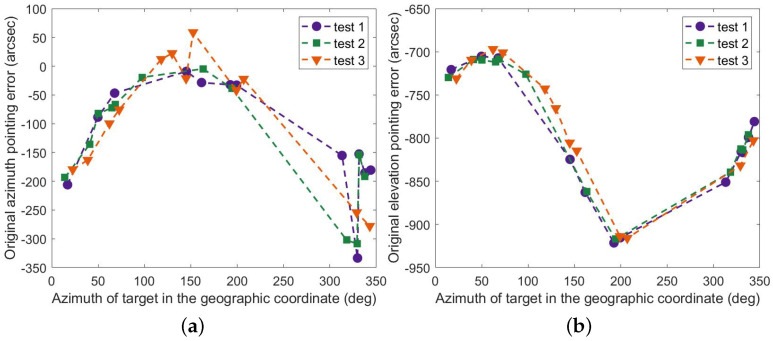
Original pointing error of the EOT (**a**) in azimuth and (**b**) in elevation.

The original pointing errors for the three subsequent tests of MPEOT can be acquired based on the theoretical positions of the target and the measured values of the encoder, as shown in Figure 11, with the errors in azimuth and elevation expressed by RMS, being 153.09″, 163.61″, 136.38″, and 813.10″, 781.03″, 789.28″, respectively. These errors are much more than 60 arc seconds while presenting certain rules.

### 5.2. Model Determination

To rectify the pointing errors, we substituted the above data into Equation (15) and solved the parameters by OLSE. The statistical analysis was then performed using SPSS Statistics, IBM, New York, USA where the VIFs of all variables exceeded 100 and some even surpassed 1000. The results indicated that the full parameter model (FPM) had severe multicollinearity that needed to be removed. Next, the stepwise regression technique was adopted to obtain the optimized parameter model (OPM) as follows, where the significance levels of entry and exclusion were set at 5% and 10%, respectively, using the F-test.

(22) $$\left\{\begin{array}{c} \delta A=\Delta \beta_{0}p_{1}+\Delta \gamma_{0}p_{2}+\Delta \alpha_{4}p_{4}+\left(\Delta A+\Delta \alpha_{2}+\Delta \alpha_{3}+k_{A2}\right)+\varepsilon_{A}\\ \delta E=\Delta rq_{5}+\Delta xq_{6}+\left(\beta_{1}-\Delta E-\Delta \beta_{4}-2\tan\left(E_{b}\right)+k_{E2}\right)+\varepsilon_{E} \end{array}\right.$$

$$\left\{\begin{array}{c} \begin{array}{cc} \delta A= & -\Delta \beta_{0}\sin\left(A_{b}\right)\tan\left(E_{b}\right)-\Delta \gamma_{0}\cos\left(A_{b}\right)\tan\left(E_{b}\right)+\Delta \alpha_{4}\sec\left(E_{b}\right)\\ & +\left(\Delta A+\Delta \alpha_{2}+\Delta \alpha_{3}+k_{A2}\right)+\varepsilon_{A}\\ \delta E= & \Delta rh_{r}\sec\left(E_{b}\right)+\Delta xh_{x}\sec\left(E_{b}\right)+\left(\Delta \beta_{1}-\Delta E-\Delta \beta_{4}-2\tan\left(E_{b}\right)+k_{E2}\right)+\varepsilon_{E} \end{array} \end{array}\right.$$

Based on the hypothesis of normality, the model has passed the *F*-test and *t*-test, indicating that the regression equation and coefficients are significant. Meanwhile, there is no multicollinearity between the independent variables as VIF≤2, which results in better robustness of the parameters estimated by OLSE. In addition, the model works well with all three criteria mentioned above, where the $C_{p}$-statistic criterion is prioritized due to the prediction purpose.

From Equation (22) we can see that the azimuth error relates closely to the angular positions of the axes. By contrast, the elevation error involves more, since $h_{r}$ and $h_{x}$ are calculated from the target position and platform attitude.

### 5.3. Experiment Results

**Figure 12:**
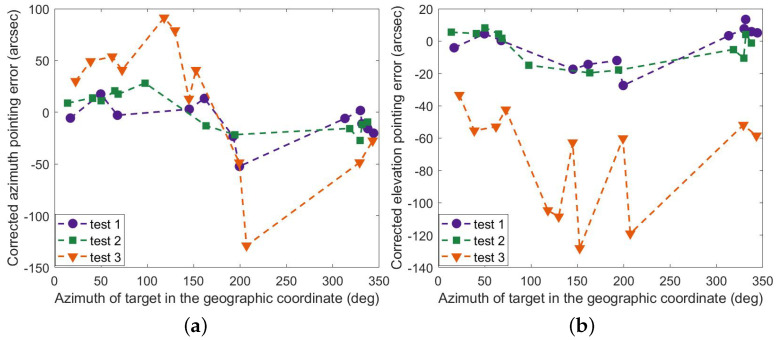
Residual errors of FPM (**a**) in azimuth and (**b**) in elevation.

**Figure 13:**
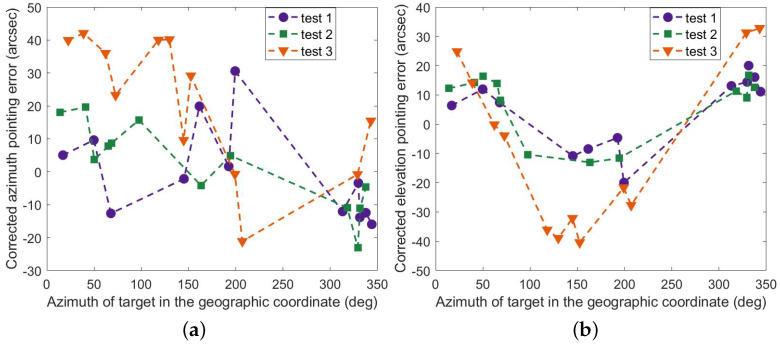
Residual errors of OPM (**a**) in azimuth and (**b**) in elevation.

**Figure 14:**
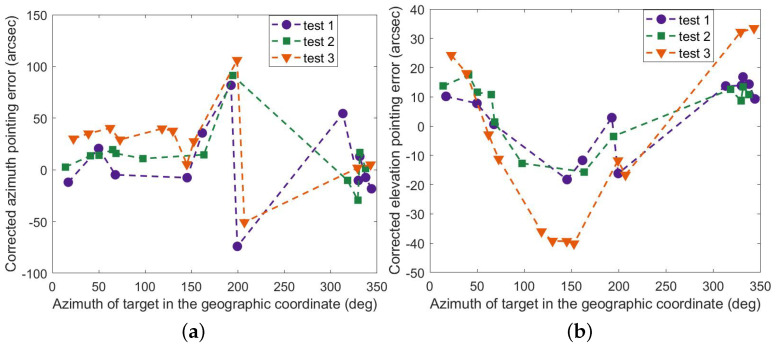
Residual errors of MM (**a**) in azimuth and (**b**) in elevation.

Figure 12 shows the results of the full parameter model (FPM) corrected for pointing errors. It is clear that the model works in a limited time, and the correction for azimuth and elevation in the third test is not effective. In contrast, the correction of the optimized parameter model (OPM) works as expected, as displayed in Figure 13, and the maximum residuals are less than 50 arc seconds in all three tests. Here are the details. The corrected azimuth (RMS) is 14.07″, 12.71″, and 28.93″, and the pointing accuracy is improved accordingly, i.e., 90.81%, 92.23%, and 78.79%. Similarly, The corrected elevation (RMS) is 12.94″, 12.73″, and 28.30″, and the accuracy is improved accordingly, i.e., 98.41%, 98.37%, and 96.41%. As seen in Figure 14, the results of the mount model are similar to those of the optimized parameter model. However, the partial residuals of azimuth fluctuate widely in all three tests.

Table 1 compares the RMS and maximum values of pointing errors corrected by the three models. Note that the results of azimuth and elevation are similar in tendency between the three experiments. In contrast, the residual curves of the azimuth and elevation are completely different in each test. What stands out in the graphs above is that there is a marked and steady trend between the revised elevation and the real azimuth of the target in the geographic coordinate, decreasing and then rising in all three trials.

**Figure 15:**
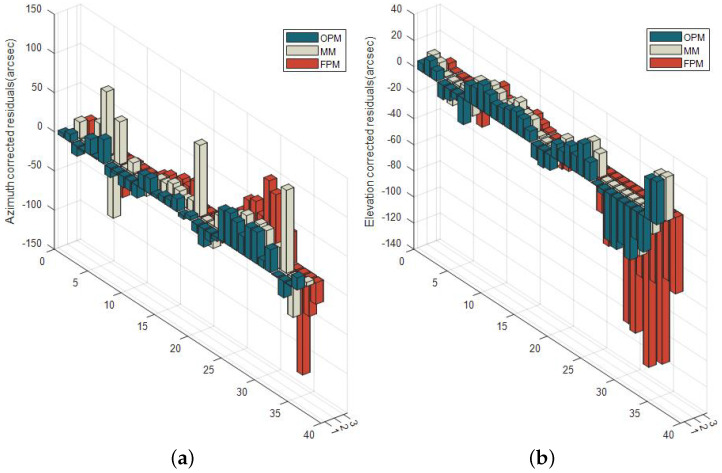
Residuals of FPM, OPM, and MM (**a**) in azimuth and (**b**) in elevation.

Figure 15 arranges these residuals in the temporal sequence of observations, divided into three groups based on the model used; each group contains twelve revised pointing errors. What can be clearly seen in this graph is that during the entire pointing correction test, a few errors from the mount model vary greatly. Importantly, the errors from the optimized parameter model have slower growing trends compared to the full parameter model, both in azimuth and elevation. Therefore, the optimized parameter model proposed in this paper can allow the MPEOT pointing to maintain a certain accuracy over a longer observation period.

## 6. Discussion

The following is a discussion of the test results. Firstly, the mount model, a critical component of modern large telescopes, was found to be ineffective for azimuth correction at certain locations in this study, while the correction effect of the two-parameter models mentioned above was better in the first two tests, especially the optimized parameter model, which performed better over a longer period. There are two possible explanations for this phenomenon: (1) The mount model only deals with mechanical errors in the device itself and not with the alignment and measurement of the platform navigation system. (2) The training samples were small and unevenly distributed, resulting in lower prediction accuracy over the range of independent variables when the model contains more relevant parameters. Secondly, although the optimized parameter model is based on kinematic analysis, the errors contained in the parameters of the model are difficult to separate, leading to reduced reliability in the identification of relevant physical parameters. This is an important issue for future research. Thirdly, the optimized parameter model performs as well as the mount model, despite having two correction terms for the elevation error. A probable explanation is that it contains multiple sources of information and no multicollinearity. Finally, the elevation residual curves in the three tests generally showed a regular trend, suggesting that a certain factor, i.e., astronomical refraction, was missed during modeling. Therefore, choosing the appropriate empirical term to compensate for this trend is another area of research interest.

## 7. Conclusions

In order to improve the pointing accuracy of MPEOT, a new optimized parameter model was proposed in this paper to compensate for the systematic errors, based on a comprehensive analysis of geometric error sources and correlation among parameters. Several star-gazing experiments with a shipboard electro-optical telescope have verified the effectiveness of the proposal.

This study has found that pointing errors involve equipment and platform navigation systems, such as manufacturing errors, misalignment, and environmental disturbance, among others. Moreover, the study found that these factors have mutually coupled effects on pointing. The second major finding was that the optimized parameter model established in this paper through stepwise regression outperformed the mount model in terms of accuracy and valid duration. Concretely, the corrected pointing accuracy was better than 50 arcsec for approximately 23 h. The presented method is also applicable to analogous two-degrees-of-freedom equipment.

## Figures and Tables

**Table 1:** Pointing precision of the EOT after correction.

Pointing Model	Experiment Number	Azimuth		Elevation	
		RMS	Maximum	RMS	Maximum
FPM	Test 1	19.67″	52.32″	12.10″	27.47″
	Test 2	17.74″	28.21″	10.12″	19.64″
	Test 3	62.18″	128.95″	79.49″	128.15″
OPM	Test 1	14.07″	30.59″	12.94″	20.02″
	Test 2	12.71″	23.02″	12.73″	16.73″
	Test 3	28.93″	42.08″	28.30″	40.42″
MM	Test 1	38.43″	81.77″	12.45″	18.23″
	Test 2	30.18″	91.25″	11.89″	17.50″
	Test 3	43.09″	106.14″	28.31″	40.15″

## Data Availability

Data underlying the results presented in this paper are not publicly available at this time but may be obtained from the authors upon reasonable request.
